# The motherhood penalty and absence of leisure rights: legal examination and institutional response under China's three-child policy

**DOI:** 10.3389/fsoc.2026.1764494

**Published:** 2026-04-07

**Authors:** Tong Huang, Zebang Hu

**Affiliations:** 1 Law School, Zhejiang Normal University Xingzhi College, Lanxi, China; 2 Law College, Macau University of Science and Technology, Taipa, Macau SAR, China

**Keywords:** fertility-friendly, gender equality, motherhood penalty, right to leisure, work-life balance

## Abstract

Under the comprehensive three-child policy, female workers face a threefold deprivation of time, capacity, and rights regarding their leisure rights, stemming from the structural tension inherent in their dual “work-motherhood” roles. The right to leisure, situated at the intersection of the right to rest, culture rights, and the right to development, constitute a core dimension for achieving holistic human development. Their jurisprudential legitimacy is rooted in the principles of substantive equality and the protection of vulnerable groups. At the level of social governance, this right serves as institutional underpinning for building a fertility-friendly society; economically, it contributes to enhancing human capital value and fostering the sustainable development of the “she economy.” To safeguard the leisure rights of female workers, a legal framework centered on “work-life balance” should be constructed. This involves core institutional measures such as paradigm reshaping, time guarantees, service support, and rights remediation, ultimately forming a synergistic e responsibility mechanism engaging the state, market, and family to promote gender equality and social sustainable development.

## 1 Introduction

Demographic transition and gender equality represent pivotal social issues in contemporary society. China's implementation of the comprehensive three-child policy to address population aging. While responding to demographic challenges, potentially reinforces inequalities arising from biological differences and socially constructed gender roles. This imposes multifaceted challenges on female works regarding career development, family responsibilities, and physical and mental wellbeing. Although supporting policies emphasize “providing robust legal safeguards” (Xinhua News Agency, 2021), they fail to address the structural dilemmas faced by women from a profound rights-based perspective. Existing research predominantly examines the impact of fertility policies on gender-based occupational advancement, with limited scholarship revealing the interactive effects of state, market, and household dynamics on women's predicament within the “work-motherhood” nexus and their corresponding rights deprivation (Minjie, 2023; Rong and Jing, 2023; Yiwei, 2024; Zhuoyan et al., 2025). While international academic has produced substantial research on “work-life balance,” its application within the Chinese context necessitates grounding in domestic realities.

The comprehensive three-child policy may systematically exacerbate gender inequality. The motherhood penalty imposes a threefold deprivation—of time, capacity, and rights—upon female workers, while the state, market and household collectively implicate them in a synergistic structural predicament. This article introduces “right to leisure” as a theoretical analytical tool. The right to leisure, intersecting the rights to rest, cultural, and development, encompasses the right to temporal autonomy, the right to choose activities, the right to accessible facilities, and the right to be free from guilt. They constitute a core dimension for citizens' holistic development. Employing a methodology that combines normative analysis with the interpretation of policy texts, this paper systematically demonstrates the theoretical basis and pathways for safeguarding female workers' right to leisure. This examination is conducted by reviewing laws and regulations, including the Constitution, the Labor Law, and the Law on the Protection of Women's Rights and Interests, alongside relevant policy documents and existing empirical research data. The objective is to provide institutional insights for building a fertility-friendly society and advancing gender equality.

## 2 Theoretical evolution: from “labor force recovery” to “holistic development”—a modern clarification of connotation of the right to leisure

The right to leisure and the right to rest are intersecting yet non-overlapping concepts, sharing similarities but also possessing essential differences in terms of legal nature, modes of exercise, and protection models. The right to leisure has not yet been established as a statutory right in China's positive law, and its extension and connotation remain uncertain due to the lack of legislative definition. Clarifying the similarities and differences between these two rights forms the theoretical basis for arguing that the right to leisure constitutes an independent right. Drawing on the normative definition of the right to rest and theories from leisure studies, the evolutionary trajectory from the right to rest, which emphasizes the recovery of labor force, the right to leisure, which is dedicated to the holistic development of the individual, can be revealed. This evolution not only reflects the expansion of the connotation of the right but also embodies the sublimation of its underlying value from an instrumental nature to a humanistic orientation.

### 2.1 The cornerstone right: the right to rest as an extension of labor rights and its limitations

Within China's current legal system, the right to rest has received constitutional-level protection. Article 43 of the Constitution stipulates:

“Working people in the People's Republic of China have the right to rest. The State expands facilities for the rest and recuperation of the working people and prescribes working hours and vacations for workers and staff.”

The right to rest is granted specifically to “working people,” indicating its inherently restricted nature of its subjects and a specific scope of application. In legal theory, the right to rest is often categorized within the framework of labor rights, regarded as a natural extension thereof, aiming to safeguard the physical health and recuperation of works. The institutional objectives of this right are not only to ensure the adequate recovery of workers' mental and physical capacities to enhance productivity but also to guarantee that workers have time to participate in political and socio-cultural life, as well as to manage family affairs (Zhiwei, 2009). The system of rest and established in China constitutes a concrete safeguard for this constitutional right.

The emergence and evolution of the right to rest are rooted in the historical interplay between labor and capital. Its institutional creation was directly motivated by the need to address the social reality of workers being over-exploited in the industrial context, ensuring them the rest time necessary for maintaining basic survival and health. This pragmatic concern has rendered the theory of the right to rest problem-oriented and characterized by pragmatism, yet it has also limited the depth and breadth of its theoretical perspective, making it difficult to transcend the specific scope of protecting the rights and interests of the worker group (Haifeng, 2022). Academic discussions predominantly focus on the instrumental value of the right to rest in restoring workers' physiological functions, generally asserting that the realization of this right helps workers recover their physical health and replenish expended energy after labor consumption, thereby sustaining their continuous labor capacity (Huai and Jia, 2023). Similarly, the interpretation of the right to rest in the Dictionary of Law emphasizes its function to “ensure the relief of workers' fatigue and the recovery and development of their physical and mental strength,” and, when classifying the right by academic discipline, implicitly categorizes it within the realm of labor law (Shourong, 2020).

In its normative orientation, the right to rest constitutes an extension and supplement to labor rights, with its institutional function focused on the objective of labor force reproduction. However, its specificity regarding the rights subject (limited to “workers”) and its pronounced emphasis on restorative functions in terms of rights content collectively constitute its inherent theoretical and practical limitations. These limitations prevent it from effectively addressing the diverse needs of broader societal members regarding comprehensive development, spiritual fulfillment, and improved quality of life.

### 2.2 Paradigm evolution: the right to leisure as a core component of the right to development

The inherent limitations regarding the subject and function of the right to rest underscore the necessity of constructing a more inclusive and developmental right to leisure. The right to rest is strictly limited to “workers,” and its exercise is confined to “periods outside prescribed working hours.” This objectively excludes non-working groups from the legitimate enjoyment of the right to rest and recuperation, leading to their structural deprivation of this entitlement. To promote the equitable distribution of time resources among all members of society and to facilitate the transformation of this entitlement from a “workers' right” to a “citizen's right,” the systematic legal justification of the right to leisure becomes an urgent theoretical task.

The right to leisure fundamentally transcends the right to rest's primary focus on physiological recovery, with its core essence lying in the promotion of human freedom and all-round development. The right to rest reflects a lower-order, restorative need, whereas the right to leisure constitutes a higher-order developmental need predicated on the satisfaction of rest, oriented toward an active state of being during free time and serving as a prerequisite for self-actualization and self-transcendence. The understanding of “leisure” encompasses a multi-dimensional theoretical spectrum: Thorstein Veblen viewed leisure as a lifestyle accessible primarily to individuals of certain social status, emphasizing that “leisure is a non-productive consumption of time.” Joffre Dumazedier, conversely, posited that

“leisure is time beyond what is necessary for survival, after obligatory tasks are completed… It is freely disposable time, utilized according to individual judgment or choice.” (Dumazedier, 1967)

Ma Huidi notes that leisure is a form of human life, on one hand eliminating physical fatigue, and on the other, providing spiritual solace (Huidi, 2019). Although Marx's theory of all-round human development does not directly address the right to leisure, it contains rich leisure-related ideas and envisions an ideal state of existence where human leisure and labor are perfectly integrated (Hairong, 2017). When humanity can free from itself from the control of capital, labor will gradually the rationality of human existence, characterizing the state of life centered around the totality of production (Tongfang, 2020). This transition from the realm of necessity to the realm of freedom enables the realization of a leap from “labor justice” to “social justice,” ultimately achieving a state of free and comprehensive development.

The legitimacy of the right to leisure can be examined within the framework of the pluralistic values pursued by law. It pertains citizens' access to justice in developmental opportunities and the distribution of social resources, reflecting the deeper value pursuit of “developmental justice.” Under the dominance of capital logic, the alienation of labor deprives workers of autonomous control over their non-working time, forming a shackle on individual comprehensive development. Establishing the right to leisure aims to restore to individuals the time monopolized by alienated labor, providing temporal guarantees for personal interests development and talent expression, thereby rectifying the substantive “entitlement deprivation” arising from individual differences. The right to leisure plays a positive role in promoting harmony within the individual and between the individual and society. A reasonable balance between labor and leisure allows individuals to experience work as an enjoyable activity engaging their physical and intellectual capacities, achieving harmony in their relationship with themselves. The degree of protection of free time reflects a society's moral boundaries; individuals require sufficient time to integrate into the process of social civilization and satisfy their deep-seated needs as social beings. The realization of human happiness constitutes a fundamental value objective of the right to leisure. By eliminating labor alienation, the right to leisure allows labor value to be fully manifested in individuals possessing adequate rest, deconstructing the paradox of labor relations under the dominion of capital logic. Only within a state of free and autonomous labor can individuals experience a sense of happiness conducive to their own survival and development.

### 2.3 Gender focus: the special significance and value of women's right to leisure from the perspective of reproduction

The strict limitations of the subject of the right to rest rends it inadequate in effectively addressing the legitimate needs of non-working groups. This institutional deficiency is particularly evident in the protection of women's rights and interests within the context of reproduction. Women who temporarily exit the labor market in response to national childbirth policies lose their status as “workers” due to unemployment, consequently making it difficult for them to invoke the constitutionally protected right to rest. However, childcare labor and household responsibilities similarly constitute a continuous drain on an individual's mental and physical energy, revealing the structural inadequacy in the coverage of the right to rest, whiling simultaneously highlighting the particular value and urgency of constructing a right to leisure for women. Examined from the perspective of reproductive, the establishment of women's right to leisure is not only crucial for the realization of individual development rights but also involves the recognition and institutional compensation for the value of social reproductive labor.

The right to leisure provides an institutional pathway for alleviating “maternal role anxiety” and promoting the realization of women's self-worth. While fulfilling reproductive responsibilities and family duties, women often face “maternal role anxiety” constructed by social expectations and role pressures, which poses continuous challenges to their physical and mental health and holistic development. As time and space autonomously controlled by the individual, leisure activities can provide women with an effective means to alleviate anxiety and pursue self-actualization. By safeguarding women's right to obtain spiritual solace and personal development during non-working hours, the right to leisure becomes a crucial support for women to break through traditional role constraints and move toward comprehensive development. From the perspective of rights compensation, the right to leisure constitutes a substantive response to women's undertaking of the social function of human reproduction. Reproduction, as a fundamental activity for maintaining societal continuity, possesses significant public good attributes (Guo and Youhua, 2019). However, when women undertake this social function, they often must sacrifice personal development opportunities and career prospects. The state and society should, through the institutional protection of the right to leisure, provide women with substantive compensation including public leisure facilities, flexible time arrangements, and specific economic support, thereby recognizing their unique contributions to social reproduction. This compensatory mechanism not only reflects respect for women's individual rights but also represents a necessary investment in the social reproduction system, demonstrating the requirements of substantive fairness and social justice in rights allocation.

The jurisprudential foundation of the right to leisure must be sought within historical and cultural dimensions. Savigny's view of the Historical School of Law posits that

“Law is by no means something that should be arbitrarily and deliberately formulated by legislators… It is rooted in the history of a nation, and its true sources are the general beliefs, customs, and the common consciousness of the people.” (Bodenheimer, 2004).

As a universal phenomenon throughout the history of human civilization, the claim for the recognition of leisure as a right aligns with the internal logic and trend of historical development. By establishing the right to leisure, achieving the equitable allocation of time resources among members of society and the reasonable expansion of the scope of rights-holders will ultimately advance the realization of the Marxist vision of the “all-round development of human beings,” thereby constructing a more inclusive and equitable paradigm for social development.

## 3 Reality analysis: the “triple deprivation” of female workers' right to leisure by motherhood penalty

Within the context of the “universal three-child” policy, the physiological and psychological pressures borne by female workers due to childbirth have been systematically exacerbated. The process of childbirth itself—from pregnancy and delivery to postpartum recovery—imposes immense physical and mental demands on women. Health risks and energy expenditures increase exponentially with multiple births. A more profound dilemma lies in the dual pressures on their time and mental resources. In the workplace, potential employment discrimination, promotion bottlenecks, and the risk of career interruption generate significant anxiety for women even during the family planning stages. Post-childbirth, they are compelled to exert effort far exceeding that of their male counterparts to compensate for career setbacks incurred. The deeply entrenched phenomenon of “motherhood penalty” often instills in women a sense of guilt toward their families while they pursue professional achievements. Furthermore, the policy environment subtly reinforces the confinement of women to the domestic sphere, thereby constricting their professional identity recognition and development opportunities.

### 3.1 Temporal deprivation: the erosion of leisure under dual labor pressures

Reproductive behavior places female workers under dual temporal pressures from the workplace and the household. This predicament is rooted in profound historical and cultural contexts and is continually exacerbated by contemporary social structures and policy environments. The deprivation of women's leisure time is the result of systemic social mechanisms. The Industrial Revolution established a clear demarcation between public and private spheres; yet, as women entered the public labor force, they were not liberated from traditional domestic responsibilities. The “second shift” dilemma burdens women with the dual load of paid work and unpaid housework. Childbirth acts as a critical turning point intensifying this plight—the “ideology of motherhood,” formed based on biological differences and social conditioning, assigns women a more central role in child-rearing, leading to a further compressing of their already limited leisure time. Marxist critiques of time reveal the alienation workers experience as capital expropriates their free time; post-childbirth female workers similarly face a severe distortion in their time allocation. According to data from the China Family Panel Studies, married women have 9.95 h of leisure per week, 7.36 h of sleep per day, and spend 3 h per day on housework. In contrast, the figures for married men are 11.17, 7.43, and 1.65 h, respectively. The weekly leisure time of married women is significantly shorter than that of married men, while their daily housework duration is nearly twice that of married men. Their time is fragmented into segments dedicated to meeting workplace demands and serving family needs, pushing leisure time essential for personal development, social participation, and physical and mental restoration to the margins. This pattern of time allocation not only impacts women's immediate quality of life but also constrains their long-term career advancement and capacity of social engagement (Table 1).

**Table 1:** Time allocation of married men and women.

Time type	Married women	Married men
Weekly leisure time (h)	9.95	**11.17**
Daily sleep duration (h)	7.36	**7.43**
Daily housework duration (h)	**3.00**	1.65

Bold values are married men and women (those who have officially registered their marriage).

In the information age, flexible work arrangements, remote work, and high-intensity work models such as “996” and “007” have blurred the boundaries between work and life. The permeability of temporal and spatial boundaries poses challenges for all demographics, yet its impact is particularly severe for women with young children. The erosion of these boundaries necessitates frequent and rapid switching between professional duties and childcare responsibilities, leading to the effective erosion of leisure time in the interstices. The implementation of the “universal three-child” policy, in the absence of adequate support systems, objectively prolongs the duration during which women remain in this high-intensity childcare phase. Consequently, there is a multiplicative increase in “invisible working hours”—time spent on childcare and housework that is excluded from economic remuneration, often eludes social statistics, and lacks institutional recognition or compensation. This unpaid reproductive labor occupies a significant portion of female workers' personal time, constituting a substantial yet frequently overlooked temporal burden.

The nature of the time deprivation mechanism is structural. Institutional deficiencies, such as the lack of family-friendly policies in labor systems and the inadequate socialization of childcare costs, interact with and reinforce the traditional gender norm of “men as breadwinners and women as homemakers.” This synergy systematically deprives women of their leisure time. This deprivation manifests not only in the quantitative reduction of available time but, more critically, in the qualitative degradation of the time that remains—women's leisure is often passive, fragmented, and contingent upon the needs of other family members, thereby undermining genuine psychological recuperation and personal development. Understanding the mechanisms behind the systematic deprivation of women's leisure time constitutes a critical entry point for comprehending contemporary gender inequality and provides a foundation for constructing more inclusive social policies.

### 3.2 Capability deprivation: the multidimensional erosion of leisure rights' viability by motherhood penalty

Leisure represents not merely a passive state of idleness, but an active, positive state of freedom. Its realization depends upon foundational supports including economic independence, cultural capital, and individual agency. The “motherhood penalty” faced by female workers due to childbearing systematically undermines their capability to realize the right to leisure across multiple dimensions. This deprivation of capability manifests not only in the economic sphere but also, more critically, in its profound impact on women's opportunities for cultural participation and their psychological wellbeing, thereby creating a self-reinforcing cycle of disadvantage.

A historical examination reveals the long-standing structural suppression of women's leisure capabilities. In feudal societies, leisure activities bore distinct class and gender attributes. Women's right to leisure sporadically existed only within specific upper strata and was often disciplined into accessories conforming to patriarchal aesthetics. The assertion that “one is not born, but rather becomes, a women” (de Beauvoir, 1998) unveils the immense power of social gendered construction—a gender gap far exceeding biological differences, which has historically widened the disparities between women and men in domains such as marriage, culture, education, and economy. If oppression of women occurs in the private sphere, then women's liberation necessarily requires them to emerge from the household, to move from the private to the public sphere (Engels, 1972). However, childbirth—a bio-social event—constitutes a significant obstacle in this transition from the private to the public sphere. Due to motherhood, female workers are more susceptible to impeded career progression, widening pay gaps, and even forced withdrawal from the formal labor market. The interruption or downward trajectory of their professional paths directly undermines their economic independence, while economic autonomy serves as the cornerstone for choosing and enjoying high-quality leisure activities.

The implementation of the “universal three-child” policy has intensified the deprivation of female workers' capabilities. Repeated childbirth signifies that multiple reproductive cycles can subject professional women's careers to successive disruptions, leading to increasingly pronounced cumulative economic disadvantages. Research by Zhang Haifeng indicates that while the adjustments in fertility policy showed no significant impact on female labor force participation, it resulted in a reduction of women's weekly working hours by 6.013 h, a decrease of 7.6% in the probability of formal employment, and a decline of 73% in wage levels. The impact of the fertility policy adjustments is particularly pronounced for women with low to medium education levels: the average weekly working hours for women with low education decrease by approximately 7.141 h, and for those with medium education by 15.211 h; for women with junior high school education or below, the probability of formal employment decreased by 8% (Haifeng, 2022) (Table 2).

**Table 2:** The impact of fertility policy adjustments on female labor.

Dimension of impact	All women	Women with low education level	Women with medium education level	Women with junior high school education or below
Impact on labor force participation	No significant impact	–	–	–
Change in weekly working hours (h)	−6.013	−7.141	−15.211	–
Change in probability of formal employment	−7.6%	–	–	−8.0%
Change in wage level	−73.0%	–	–	–

The deprivation of capability manifests not merely as material constraints, but more profoundly as a systemic erosion of intrinsic developmental capacities. The mental exhaustion resulting from chronic time poverty and role overload—simultaneously fulfilling the roles of employee, mothers, and homemaker—often leaves women, even during rare moments of leisure, too depleted to engage in energy-demanding, developmentally meaningful leisure practices. Consequently, they are often relegated to passive, low-quality forms of recreation. This systematic deprivation of leisure capabilities is not only a matter of individual wellbeing but also reflects deeper social inequities. It impedes the fundamental right of women, as full social subjects, to achieve self-realization and foster comprehensive development through free and conscious leisure activities.

### 3.3 Rights deprivation: performance bias, intensive motherhood, and the absence of legal protection

Reproductive behavior renders female workers more susceptible to the dual pressures of workplace performance bias and the cultural stigma of “perfect motherhood,” leading to the systematic deprivation of their leisure rights at a structural level. Within the current legal system, while the right to rest is established as a positive legal concept, the right to leisure demands a higher-order positive liberty encompassing both free time and autonomous activities—a right difficult to realize in practice due to gendered social mechanisms. Research by Wang Meiyan finds: compared to unmarried women, married women are 16% less likely to participate in the labor market; compared to unmarried men, married men are 12.3% more likely to participate; women with children aged 0–6 are 14.99% less likely to participate than those without; and men with children aged 0–6 are 3.5% more likely to participate than those without (Meiyan, 2023a). The workplace stereotype branding childbearing women as “low-efficiency workers” directly results in their marginalization in promotions, core task assignments, and salary increases. To counter this bias and maintain professional competitiveness, women are compelled to overdraw their already limited energy, thereby allowing workplace performance pressures to encroach upon the physical and mental space essential for leisure (Table 3).

**Table 3:** Change in probability of labor force participation by group comparison.

Group comparison	Change in probability of labor force participation (%)
Women with spouse vs. women without spouse	−16.0
Men with spouse vs. men without spouse	12.3
Women with children aged 0–6 vs. women without children	−15.0
Men with children aged 0–6 vs. men without children	3.5

The cultural phenomenon and moral discipline surrounding the “perfect mother” in society impose invisible shackles upon women (Meiyan, 2023b). It constructs the maternal role as a child-centric, self-sacrificing ethical mission, thereby placing women's leisure activities under moral scrutiny—any investment in personal pleasure and development may be condemned as a deviation from maternal duties. Leisure ceases to be a right and instead becomes a source of “guilt” that must be overcome, its realization hindered by internalized moral anxiety. Within the male-dominated social power structure, women often occupy disempowered, marginalized, and structurally weakened positions, a situation further exacerbates by childbirth. This rights deprivation manifests not only at the individual level but also reflects deeper institutional flaws: the current labor legislation system lacks effective mechanisms to identify de facto discrimination, the social security system fails to fully recognize the social value of reproduction, and public policies remain inadequate in promoting the shared responsibility of family duties.

Faced with systemic rights deprivation, the law ought to undertake the responsibility of assisting the vulnerable. While the law is not inherently a tool to eradicate power imbalances, it must shoulder the duty of supporting the disadvantaged… The law cannot stand idly by when confronted with the plight of the weak (Yuhong, 2021). However, under the adjusted “universal three-child” fertility policy, court data indicate that employers, driven by economic efficiency, frequently opt to unilaterally terminate labor contracts and provide compensation when dealing with female employees on special leaves (e.g., pregnancy-related sick leave, maternity leave, parental leave). For instance, the Beijing Third Intermediate People's Court handled 188 labor dispute cases from 2022 to 2024 involving special leaves for women, accounting for 3.8% of its caseload. Similarly, the Suzhou Intermediate People's Court's “White Paper on Safeguarding Women's Rights and Interests (2022–2024)” reported 13,047 labor dispute cases involving female employees, constituting 24.06% of all labor disputes accepted. These cases often relate broadly to the female employment environment, such as protections for reproductive rights and workplace gender discrimination. Judges have emphasized that employers should prioritize the rights and interests of special groups, fostering a corporate culture with humanistic care and a well-ordered environment for employee career development. Despite these rulings, the state of “rights impoverishment” that women fall into due to childbirth has not been effectively alleviated. The right to leisure remains generally overlooked in policy discussions and institutional design. This neglect is not only a matter of individual quality of life but also serves as a crucial benchmark for testing whether the law genuinely fulfills its promise of gender justice.

## 4 Jurisprudential justification: the multifaceted legitimate foundation for safeguarding female workers' rights to leisure

The legitimacy of protecting female workers' right to leisure is rooted in a multidimensional jurisprudential framework. Its significance extends far beyond individual welfare claims, touching upon the core values and future trajectory of a modern society governed by the rule of law. The jurisprudential justification for the right to leisure must transcend superficial analysis and delve into the underlying values, legal foundations, social functions, and economic rationales that support it. This protection is not only an essential requirement for realizing substantive gender equality under the constitution but also a crucial component in responding to national population development strategies and fostering high-quality economic development.

### 4.1 Value foundation: the legal logic based on substantive equality and protection of vulnerable groups

The value foundation for safeguarding female workers' right to leisure is rooted in the evolution of constitutional principles in modern rule-of-law states, which have progressed from “formal equality” to “substantive equality.” This principle requires the law to pay attention to the specific circumstances of different groups and provide necessary preferential protection to female workers who are structurally disadvantaged due to childbirth. The paradigm of legal philosophy research in China has undergone a profound shift from the “class struggle paradigm” to the “rights-based paradigm.” This transformation emphasizes respect for the human subject as the core of law, allowing human autonomy, self-awareness, self-direction, and self-discipline to be fully expressed through the fundamental particles of law - the relationship between rights and obligations (Wenxian, 2001). The right to leisure reflects respect for the comprehensive development of the individual and is a manifestation of the “rights-based paradigm” in the content of specific rights.

The establishment of the right to leisure aligns with the constitution pursuit of substantive justice. Adherence solely to “formal equality,” while disregarding the unique physiological burdens imposed on women by childbirth and the social constraints arising from the traditional gender role norms of “men as breadwinners and women as homemakers,” would effectively perpetuate existing inequalities. The establishment and protection of women's right to leisure constitute a necessary requirement for the law to implement the principle of “fairness for the disadvantaged” and to redress gender-based disparities. This concept resonates with feminist philosophical tenets, which advocates for women to fully utilize potential forms of collective struggles to overthrow the entire system of domination (Qing, 2018). Safeguarding the right to leisure precisely challenges the traditional paradigm that confines women to the private sphere, empowering them with the legal capacity to control their own time and participate in public life.

The Communist Party of China's historical endeavors in advancing women's emancipation have furnished localized political and ideological foundations of the liberating essence inherent in the right to leisure. Since the formulation of the “Resolution on the Women's Movement” in 1922, the Party initiated women's liberation practices during the Soviet Ruijin era, which underwent substantial developed throughout the Yan'an period. In 1939, Mao Zedong articulated at the Yan'an Women's Congress: “To genuinely attain emancipation, we must mobilize the extensive ranks of women to participate.” The monthly publication Chinese Women, established during this epoch, exerted a pivotal influence in propagating the Party's policies concerning women and fostering feminist consciousness within revolutionary base areas (Xian'e and Xiaoyao, 2021). Central to these initiatives was the conceptualization of women's liberation as an integral component of social revolution, underscoring the achievement of women's self-emancipation through engagement in social production and public affairs.

The aforementioned theory and practices are ultimately rooted in Karl Marx's ultimate ideal of human emancipation. Marxism pursues the free and comprehensive development of individuals, wherein the control over free time is instrumental in achieving such development. Safeguarding women's right to leisure constitutes an essential pathway to liberating them from alienated labor and entrenched gender roles, thereby facilitating their comprehensive development. The law bears the responsibility of supporting the disadvantaged and, through the principle of “equity for the disadvantaged,” rectifying historical and structural inequities. This establishes a foundation value for their genuine and equal participate in social life and realization of personal potential, reflecting the profound commitment of socialist rule of law to substantive justice.

### 4.2 Juridical basis: the intersection and integration of the right to rest, cultural rights, and the right to development

As an emerging right, the legitimacy of the right to leisure emanates from its intrinsic alignment with and integration into the modern system of rights, representing the confluence of three major rights categories—the right to rest, culture rights, and the right to development—and demonstrating a natural logic in the evolution of rights. Within the Hohfeldian analytical framework of rights, the right to leisure exhibits a multifaceted juridical structure: it encompasses the individual's “liberty” (or “privilege”) to demand that the state or third parties refrain from interfering with their lawful leisure activities; it entails the “claim” right to demand that the state provide necessary leisure facilities and conditions; it also pertains to the “power” right to receive equal treatment within the realm of leisure; and it involves the “immunity” from having one's leisure opportunities unduly deprived. This refined juridical structure can be articulated through five specific legal competencies:

Autonomy in Time Allocation embodies the “liberty” within the Hohfeldian framework wherein individuals possess the right to freely dispose of their non-working time while the state or third parties bear a correlative duty of non-interference without justification. The right to choose activities is another manifestation of “freedom,” where individuals have the autonomy to select their leisure activities without any coercion. Freedom of Activity further reflects this “liberty,” guaranteeing the individual's autonomy in selecting forms of leisure free from coercion. Right of Access to Facilities imposes upon the state a positive obligation to provide public leisure facilities and conditions, thereby ensuring equitable and convenient access to leisure resources. The Right to Capacity Development integrates both “claim” and “power” dimensions, requiring the state not only to establish conditions for self-actualization through leisure but also to endow individuals with the legal capacity to alter their jural relations within this domain. Correspondingly, the Right to Freedom from Stigma aligns with “immunity,” serving to safeguard individuals against adverse workplace treatment or unwarranted social moral disapproval arising from their engagement in leisure activities.

The right to leisure represents the necessary evolution of the right to rest under modern social conditions. The right to rest, as stipulated in China's Constitution, primarily focuses on the reproduction of labor power, guaranteeing the fundamental need for workers' physical recuperation. Building upon this foundation of physiological recovery, the right to leisure further emphasizes individual self-development and enhancement of life quality through Autonomy in Time Allocation and Freedom of Activity Choice during freely disposable time. This evolution embodies the progression of rights philosophy from a “subsistence-oriented” to a “development-oriented” paradigm.

Cultural rights imbue the right to leisure with spiritual significance. In accordance with international human rights conventions and relevant Chinese legislation, citizens enjoy the right to participate in cultural life. The right to choose cultural activities and the right to develop cultural capabilities transform abstract cultural rights into concrete cultural practices. The right to access cultural facilities provides the material foundation for cultural participation. For female workers, the right to leisure directly determines the level of realization of their rights to cultural participation and cultural expression, thereby influencing the richness of their spiritual world and the integrity of their personality development.

The right to leisure, viewed through the lens of the right to development, entails profound significance. The right to development emphasizes holistic individual progress across economic, social, culture, and political dimensions. The right to temporal autonomy and the right to capacity development provide essential temporal guarantees and developmental space for personal growth. The right to leisure free from guilt holds particular value for female workers in vulnerable position due to childbirth. By eliminating moral obstacles to leisure, it helps women overcome work -procreation role tension and achieve comprehensive personal development.

Within the increasingly robust framework of international human rights discourse, the principle that “women's rights are human rights” has gained universal recognition. Explicitly incorporating the right to leisure into the scope of legal protection not only complements and synergizes with existing rights such as the right to rest, culture rights, and the right to development, forming an interconnected network of rights, but also tangibly enhances women's agency through the realization of five specific entitlements. The establishment of the right to leisure signifies society's profound recognition of the need for holistic individual development and reflects the progression of the rule of law from formal to substantive equality.

### 4.3 Social foundation: rights-based support for building a birth-friendly society

The protection of women's right to leisure constitutes an inherent requirement for achieving sustainable social development, carrying particular practical significance during China's current demographic transition. While the comprehensive three-child policy implemented in China aims to optimize demographics structure and promote long-term balanced development, its effectiveness largely depends on the extent to which women's reproductive autonomy is safeguarded. Neglecting the protection of women's right to leisure during policy implementation would lead to excessive concentration of childbearing costs on women themselves. This would not only undermine the policy's intend outcomes but also potentially exacerbate gender inequality. Chinese society currently faces the dual challenges of boosting fertility rates and promoting gender equality—two objectives that are intrinsically aligned and require organic integration through institutional innovation.

Contemporary women universally face the paradoxical predicament of “childbearing vs. career development”: despite its crucial contribution to societal population reproduction, childbearing often leads to practical consequences including career interruption, income reduction, and constrained personal development opportunities. The high opportunity cost of childbearing has become a key factor suppressing reproductive willingness. Resolving this dilemma requires constructing a genuinely birth-friendly society. The realization of a birth-friendly society must center on comprehensive human development, encompassing physical and mental health, satisfaction of needs, enhancement of capabilities, and harmonious coexistence between humanity and nature (Wenxing, 2021). From this perspective, the right to leisure directly contributes to enhanced reproductive willingness by improving women's quality of life, alleviating parenting pressure, and promoting personal development, thereby creating favorable conditions for effective population policy implementation.

From a historical perspective, women's leisure conditions demonstrate a significant positive correlation between societal openness. The breadth and depth of leisure activities participated in by Tang Dynasty women reflected, to a certain extent, the openness and vitality of society at that time (Xiaofang, 2020). Within a relatively liberal social environment, Tang Dynasty women could engage in diverse leisure activities, which not only manifested the social prosperity of the era but also provided certain developmental spaces for women. This historical evidence indicates that the level of protection of women's right to leisure serves as an important measuring of social progress and civilization.

In the contemporary social context, systematic institutional design is required to establish a comprehensive leisure support system. This system should include: strengthening the provision of public leisure services to ensure accessibility of leisure resources; improving childcare support policies to alleviate women's care burden; and fostering a socio-cultural environment that respects leisure, thereby eliminating prejudice against women's leisure. Through these multi-pronged institutional safeguards, women will be better equipped to reconcile and balance reproductive responsibilities with personal development, ultimately achieving a virtuous cycle between population development and social progress. In this process, the right to leisure serves not only as a guarantee for individual wellbeing, but also importantly as a crucial link connecting population policies with gender equality, playing a foundational role in building a sustainable society.

### 4.4 Economic foundation: the dual effects of human capital appreciation and “she-economy” activation

The protection of women's right to leisure carries significant economic implications, primarily manifested through two key dimensions: enhancing the human capital quality and stimulating market consumption vitality. At the micro level, moderate leisure engagement enables female workers to effectively mitigate occupational burnout while promoting physical and mental health, thereby maintaining and improving work performance. Management research indicates that scientifically balanced leisure activities not only alleviate work-related stress but also stimulate innovative thinking and professional enthusiasm. When adequate leisure safeguards are institutionalized for female workers, their labor productivity and work quality demonstrate substantial improvement—an outcome of considerable importance for enhancing overall economic efficiency. In the knowledge—driven economy where human capital constitutes a core element of economic growth, protecting women's right to leisure essentially represents a strategic investment in high-quality human resources.

At the meso level, a virtuous interaction has been formed between the protection of the right to leisure and the development of the leisure industry. With the enhancement of women's economic status and consumption capacity, the “She Economy” has emerged as a significant force driving the optimization of the economic structure. The release of female leisure demand directly stimulates the development of multiple industries, including cultural entertainment, fitness and tourism, and education and training, thereby creating new economic growth points. Relevant statistical data indicates that women's proportion in leisure consumption has been steadily rising in recent years, establishing them as a major driver propelling the growth of the leisure industry. This transformation of the consumption pattern not only reflects the improvement in women's economic status but also demonstrates the economic benefits arising from the realization of the right to leisure.

At the macro level, the protection of the right to leisure contributes to rectifying resource misallocation under market mechanism. The development model solely focused on economic growth tends to generate a propensity to alienate workers into mere instruments of productions. Safeguarding the right to leisure serves as a corrective measure against such alienation, ensuring workers' entitlement to autonomous development space and thereby harmonizing economic objectives with the goal of comprehensive human development. This integration not only aligns with the fundamental requirements of the socialist market economy but also represents a crucial pathway toward achieving high-quality economic development.

Within the context of the comprehensive three-child policy, the economic significance of protecting the right to leisure becomes increasingly prominent. By mitigating the negative impact of childbearing on women's career progression, the safeguarding of leisure rights helps enhance female labor force participation rates and employment quality, thereby maximizing the economic benefits of population policies. Simultaneously, the protection of the right to leisure facilitates the unleashing of women's consumption potential, injecting new momentum into the domestic economic circulation. These dual economic effects demonstrate that safeguarding women's right to leisure not only carries social value but also constitutes a vital measure for promoting sustainable economic development.

The realization of economic benefits from the protection of leisure rights requires a systematic policy framework. This entails strengthening the labor legal system to ensure effective safeguarding of women's leisure rights, developing diversified leisure industries to meet the recreational needs of different female demographics, and fostering an equitable social environment to eliminate various barriers to women's leisure participation. Only through such comprehensive measures can the economic benefits of leisure rights protection be fully realized, thereby achieving positive interaction between economic development and holistic human development.

## 5 Pathway exploration: constructing a legal safeguard system for “work-life balance”

China currently stands at a critical juncture of population policy transforming and deepening rule of law construction. While the comprehensive three-child policy aims to optimize demographic structure, it simultaneously reveals the systemic tension women face in balancing career development, family responsibilities, and leisure realization. Due to physiological differences and sociocultural gender norms, women have long maintained a relatively disadvantaged position, with reproductive behavior imposing additional burdens and rights dilemmas. Therefore, establishing a legal protection framework centered on the right to leisure demonstrates not only institutional necessity but also represents a crucial pathway for advancing gender equality and promoting holistic human development. Moreover, it serves as a critical focal point for achieving coordinated advancement between population policies and social policies.

### 5.1 Guiding principles: reconstructing the roles of state, market, and family through balanced development and shared responsibilities

Within the framework of the comprehensive three-child policy, establishing a protection system for women's right to leisure fundamentally requires the adoption of two conceptual objectives: “work-life balance” and “shared societal parenting.” As the transformation of population policies advances, the structural contradictions women face in balancing career development, family responsibilities, and personal leisure have become increasingly prominent. Traditional sociocultural gender norms often implicitly designate childcare and domestic labor as women's “natural duties,” with reproductive behavior further exacerbating inequalities in time allocation and role undertaking (Xiaoying, 2018). Aiming for “work-life balance” necessitates a fundamental shift from the traditional development paradigm that prioritizes “efficiency” and “output” above all else. Labor system dominated by market economic logic frequently overlook the unpaid contributions of workers, particularly women, in household reproduction, thereby trapping them in the dual predicament of “time poverty” and “role overload.” The state must transition from passive respect to proactive engagement, legally affirming the right to leisure as a fundamental human right through legislation, incorporating it into the protective scope of labor law and social security law, and establishing a tripartite responsibility-sharing mechanism among the state, market, and family—tailored to China's national context—through institutional designs such as flexible working arrangements, parental leave, and paid vacation systems.

In the practice of balancing these interests, the rule of “dynamic balance between efficiency and equity” should be followed to appropriately address the tension between enterprises' operational needs and women's right to leisure. It is essential to recognize the rationality of enterprises in pursuing production efficiency, while also associating the protection of women's leisure with the sustainable development of enterprises through institutional design. For instance, the designation of “family-friendly enterprises” can enhance their competitiveness in the talent market, thereby transforming the protection of women's right to leisure from a “cost burden” into an investment in “human capital.” Furthermore, economic instruments such as tax reductions and exemptions, as well as social insurance subsidies, can alleviate the increased operational costs that enterprises may incur due to the implementation of flexible working arrangements.

The concept of “shared society parenting” aims to break the traditional rule that indoctrination that “motherhood is a natural destiny,” redefining childrearing as a public undertaking possessing social reproduction value. While China's existing legal framework already incorporates maternity leave, breastfeeding breaks, and related institutions, challenges persist including singular responsibility attribution and insufficient male participation. Childrearing should not constitute a solo performance of mothers, but rather a structural project requiring collective societal engagement (Henderson et al., 2000). By expanding the provision of public childcare services and developing inclusive early education institutions and community-based parenting support centers, a multi-tiered childcare service system characterized by “state guidance, market supplementation, and community support” shall be established (Shirong, 2000). Building upon existing legislation, it is imperative to further strengthen paternal childcare responsibilities through fiscal and social security measures that incentivize gender-egalitarian division of labor within households.

The state should strengthen legislative safeguards and policy coordination by incorporate the protection of the right to leisure into national development strategies. Through enacting an Anti-Employment Discrimination Law or revising the Labor Law, relevant provisions concerning “work-life balance” should be established to clarify the employers' statutory obligations. Enterprises and other employing entities must substantively fulfill their “family-friendly” social responsibilities by recognizing the long-term value of employee leisure and wellbeing for sustainable human capital accumulation. Employers may implement telecommuting and flexible working arrangements to alleviate employees' work-family conflicts. The government may provide tax incentives, targeted subsidies, or preferential treatment in public procurement to enterprises demonstrating exemplary compliance. Within domestic spheres, cultural guidance and institutional incentives should reconstruct the division of parental roles. Societal expectation of “idealized motherhood” constrict women's leisure and development opportunities. There should be active promotion of equitable sharing of childrearing and household duties within families, driving transformative changes in gender role perceptions through institutional means.

### 5.2 Model selection: constructing a multi-tiered and comprehensive legislative framework for the protection of the right to leisure

As one of the Constitutional social rights, the right to leisure constitutes an “incomplete subjective right,” the realization of which cannot be directly sought through judicial recourse, but depends on the positive acts of the State and is constrained by factors such as regional development levels, fiscal capacity, and gender equality. The reality of uneven regional development in China determines that women's right to leisure cannot be protected with uniform standards and consistent content across all regions of the country.

The value of the right to leisure lies in shaping an objective value order within the social community, which requires that the exercise of legislative, executive, and judicial powers at both the national and local levels must uphold and practice it. Even if individuals rights awareness has not yet been awakened, or certain regions are constrained by limited conditions, the state and local authorities remain obligated to take positive actions in areas such as resource allocation, institutional construction, and budgetary guarantees to ensure the realization of the minimum standard of the right to leisure. To put this objective value order into practice and effectively advance the transformation of female workers' right to leisure from concept to reality, China should adopt a multi-tiered and comprehensive legislative protection model. Through the state's positive actions, this model aims to address the challenges posed by uneven regional development and solidify the baseline for the protection of women's right to leisure.

Within the labor law system, the institutional foundation for the right to leisure must be further consolidated. Although existing legislation such as the Labor Law and the Labor Contract Law regulate working hours, rest periods, vacations, and overtime restrictions, they have not yet fully recognized the independent value of the right to leisure in their conceptual orientation. Future legislative amendments should promote a purposive expansion interpretation of the “right to rest,” extending its connotation from physical recovery to holistic personal development. In specific institutional design, building upon existing rigid provisions such as maximum working hours, paid leave, and overtime restrictions, particular attention should be paid to protecting female workers' leisure during special periods. This includes implementing flexible working arrangements during childcare periods and accumulating overtime hours for compensatory leisure leave, thereby creating economic disincentives for employers to encroach upon female workers' leisure time (Yinan, 2022).

Through the special law system, targeted protection for female workers' right to leisure should be established. Legislation such as the Family Education Promotion Law and the Law on the Protection of Women's Rights and Interests provides legal anchors for this purpose. It is recommended to elaborate provisions on family responsibilities and gender equality, explicitly affirm the state's obligation to guarantee female workers' right to leisure, and create conditions for its enjoyment and exercise. Local governments should alleviate parenting pressure and create leisure opportunities for female workers by providing temporary childcare services through measures such as purchasing services.

Given the uneven regional development in China, the protection of female workers' right to leisure must be tailored to local conditions. In areas with limited resource endowments, it is inappropriate to simple apply the protection standards of developed regions. A differentiated model of “basic guarantees + supplementary features” should be established in light of local circumstances: less developed regions should first ensure the regions implementation of core institutions such as statutory working hours and leave; on this basis, economically developed regions are encouraged to further specify governmental responsibilities in constructing leisure facilities and organizing public recreational activities. Drawing from childcare subsidy schemes already implemented in certain areas, it is essential to effectively reduce both the temporal and economic burdens of childrearing on families. By improving public service system, the material foundation for realizing the right to leisure shall be established (Yanjun, 2021). The financial pressure on less developed regions in implementing birth security and constructing public leisure facilities should be alleviated through special central government funds or transfer payments, ensuring that women in different regions can enjoy a basically homogeneous baseline of protection for the right to leisure.

The establishment of a monitoring and evaluation mechanism is crucial for ensuring legislative effectiveness. Legislation should explicitly assign the State Council Working Committee on Women and Children the regularly responsibility for assessing the protection status of female workers' right to leisure across regions, accompanied by the issuance of specialized reports. Social organizations and public participation in supervision should be encouraged, forming a synergistic force between public authority and social oversight (Wei, 2020).

Through multi-layered legal intervention, the structural imbalance in gender power dynamics shall be adjusted to transform women's status of rights deprivation (Yuhong, 2021). By progressively establishing a gender-friendly system for the protection of leisure rights, the advancement of social fairness and justice shall be promoted. The equal enjoyment of the right to leisure serves as a crucial indicator for measuring gender equality, while also constituting an indispensable condition for achieving comprehensive human development.

### 5.3 Core mechanisms: establishing a tripartite system centered on time security, service support, and rights remediation

To systematically address the structural barriers female workers face in the realm of leisure, the primary focus must be on implementing a time security system to dismantle the temporal constraints hindering the realization of their right to leisure. This entails nationwide implementation of paternity leave with clearly defined duration and salary standards, thereby promoting intra-household sharing of childcare responsibilities. The maternity leave system requires enhancement with strengthened enforceability. While appropriately extending paid maternity leave, it is crucial to ensure its effective implementation in practice. Establishing a social cost-sharing mechanism for maternity leave through the creation of a maternity security fund through the creation of a responsibilities within families. Improve the maternity leave system and enhance its rigidity. While moderately extending paid maternity leave, it is necessary to effectively increase its implementation rate. Establish a social cost-sharing mechanism for maternity leave, set up a maternity security fund would alleviate employers' cost concerns associated with female employees' leave and prevent the indirect marginalization of women due to childbirth (Jingyi and Zhu, 2021). Furthermore, the right to request flexible working arrangements should be established and specified, permitting female workers with young children to apply for reduced hours, telecommuting, or flexible scheduling. Concurrent anti-discrimination arrangement of working start and end times. At the same time, anti-discrimination provision must safeguard female workers from adverse treatment in promotion, compensation, or training opportunities due to exercising their right to leisure.

When implementing flexible work arrangements, full consideration should be given to industry characteristics and enterprise scale, adopting differentiated implementation forms. For instance, manufacturing and service industries may adopt the “hours banking” model, which allows female workers to flexibly allocate working hours within specific period around childbirth, accumulating “working time credits” during peak seasons and redeeming them for leave during off-peak seasons or when family care is needed. Professional service industries may adopt a “core working hours + flexible commuting” model, specifying a mandatory online period each day, while the remain working hours are arranged by employees themselves, with work outcomes serving as performance evaluation indicators. Additionally, for small and medium-sized enterprises (SMEs), which have limited capacity to bear management costs, the government may provide support through the procurement of services, such as outsourcing human resource management, to lower the threshold for implementing such arrangements.

When refining the mechanism for shared parenting responsibility, large enterprises should be encouraged to establish “paternity leave” provisions that are mandatorily linked to performance evaluations. It shall be clearly stipulated that paternity leave is exclusively reserved for fathers and is non-transferable. Concurrently, “family-friendly” social insurance subsidies should be granted to enterprises employing women of childbearing age, serving as compensation for the managerial costs incurred when employees exercise their rights to flexible work arrangements. Furthermore, in regions with insufficient childcare resources, female workers with young children shall be permitted to adopt flexibly working arrangements such as reduced working hours or telecommuting. Such provisions must be accompanied by anti-discrimination regulations, ensuring that female workers do not suffer adverse treatment in promotions, remuneration, training opportunities, or other aspects of employment by reasons exercising their right to leisure.

Strengthening diversified service support mechanisms to establish public infrastructure for the realization of female workers' right to leisure. As the primary provider of public services, the state must alleviate the unpaid care burden on female workers to create conditions for their leisure participation. Concurrently, substantial efforts should be directed toward developing an inclusive childcare service system, expanding its coverage and enhancing service quality. Furthermore, advancing the construction of community-based public cultural service facilities—such as libraries, community centers, and green parks—is essential to provide women with accessible and available leisure spaces and activity resources. Supports should be extended for the implementation of public-interest cultural, sports and educational training programs tailored to women, thereby enhancing their leisure capabilities and quality of life. Through comprehensive service provision, the state facilitates the socialization of reproductive labor costs.

Establish an effective rights remedy system to ensure female workers' right to leisure transition from normative entitlement to substantive realization. At the administrative level, violations such as infringing of rest and vacation rights and denial of reasonable flexible work arrangements must be explicitly incorporated into the scope of labor supervision. Labor inspection authorities should enhance proactive monitoring of such illegal acts, thereby strengthening public authority protection for leisure time entitlements. Judicially, it is imperative to refine relevant evidential burden rules in labor dispute arbitration and litigation. Recognizing workers' disadvantaged position in evidence provision, the burden of proof should be appropriately reduced for workers in disputes involving adverse treatment, with partial evidential responsibilities shifted to employers under specified conditions. Concurrently, exploration should be made to incorporate “leisure interests infringement” within the purview of anti-discrimination or matrimonial family law. In cases of workplace indirect gender discrimination, any measure or regulation that systematically results in female workers bearing excessive domestic burdens thereby depriving them of reasonable leisure may constitute discriminatory practice. In divorce proceedings, circumstance where female workers' developmental opportunities have been constrained due to long-term assumption of excessive household obligations, resulting in leisure deprivation or insufficiency, should be considered as factors for domestic labor compensation. This approach enhances legal responsiveness to structural and implicit inequalities.

### 5.4 Coordination mechanisms: establishing a multi-stakeholder governance framework involving policy guidance, market incentives, and social participation

The legal protection of work-life balance cannot rely solely on legislative mandated, but requires the construction of a set of coordination incentive mechanisms that guide market and social forces to share responsibilities collectively. This synergistic approach leverages policy resources to stimulate market vitality, thereby forming an organic system characterized by positive interaction among policy, market, and society.

The state's adoption of incentive-based policies to guide employers in implementing family-friendly measures has become a crucial pathway for advancing work-life balance. In recent years, numerous regions across China have actively explored policy models that link flexible work arrangements and childcare support initiatives with tangible corporate benefits. For instance, in November 2023, Shanghai launched a city-wide pilot program for “Fertility-Friendly Positions,” encouraging employers to designate roles with flexible scheduling options as such positions for workers responsible for children under 12 years of age. Participating enterprises receive prioritized support in areas including employment services, business incubation bases, and eligibility for advanced enterprises selection awards. Shanghai's “Fertility-Friendly Positions” initiative aims to establish a universal employment model that harmonizes corporate efficiency with social value.

In addressing the relationship between temporal autonomy and enterprise operational needs, it is essential to properly balance the cost—benefit dynamic. Regarding enterprises' concerns about rising management costs due to flexible work arrangements, a cost socialization approach may be adopted. The government could provide a certain percentage of subsidies through procurement of services or tax credits, thereby transforming enterprises-borne costs into socially shared costs. With respect to regional resource disparities, developed regions may take the lead in constructing public leisure facilities and inclusive childcare systems, thereby providing solid material support for temporal autonomy. Less developed regions, on the other hand, may leverage social capital mobilization mechanisms such as “time banking” and “community mutual assistance” to safeguard women's basic disposable time. Furthermore, it should be clearly stipulated that the exercise of the right to flexible work shall not impede the normal production and operation of enterprises. When employees request flexible work arrangements, enterprises may negotiate specific alternative solutions with the employees.

Integrating multi-sector resources to establish a coordination inter-departmental mechanism for generating synergistic promotion of work-life balance. The active participation of social organizations such as trade unions and women's federations provides both internal impetus and external oversight for work-life balance initiatives. Through collective bargaining and rights advocacy, these organizations promote the incorporation of female workers' leisure rights into labor contracts, thereby ensuring the realization of such entitlements. The Guidelines on Promoting Compliance Awareness and Strengthen Compliance Management in Small and Medium Enterprises, jointly issued by the General Offices of 15 ministries including the Ministry of Industry and Information Technology, explicitly state:

“Safeguarding employees' lawful rights and interests, including receiving remuneration, enjoying rest periods and vacations, participating in social insurance, and undergoing vocational training.”

This provision offers a policy basis for the active engagement of trade unions and women's federations. Jiangxi Province has explored the establishment of a “Four 100s” cultural mentorship program directly serving grassroots mechanism. Launched in February 2025, this initiative focuses on four key areas—theoretical dissemination, journalism, arts, and intangible cultural heritage—aiming to optimize the supply of cultural products and services, infuse industrial development with greater cultural significance, enhance development quality, and achieve a win-win effect outcome of cultural and economic value.

Strengthening public education and enhance societal recognition of female workers' right to leisure constitutes an essential safeguard for ensuring effective legal implementation. The right to leisure represents not only individual entitlement but also an indicator of social progress and civilization. Achieving work-life balance necessitates corresponding sociocultural foundations. Shanghai's “Fertility-Friendly Positions” initiative should transcend being merely a special category of employment to become an environment guided by institutional and cultural frameworks. Beyond utilizing policies to direct employers, it is imperative to cultivate corresponding corporate culture and social atmosphere. The media should fully leverage its role in shaping societal perceptions by enhancing public awareness and acceptance of work-life balance policies, thereby fostering a societal consensus that supports the protection of leisure rights and promoting a virtuous development of the cultural environment for work-life balance.

## 6 Conclusion

Leisure represents one of the significant social issues confronting our era (Guangyuan, 2005), whose realization directly impacts individual wellbeing and social equity. While a “leisure class” has emerged in contemporary China, for the vast majority of female workers, leisure remains an inadequately realized right. The 19th National Congress of the Communist Party of China identified the “contradiction between unbalanced and inadequate development and the people's ever-growing needs for a better life” as the principal societal challenge. The unequal realization of leisure rights embodies this contradiction in practical terms. Advancing the legalization and institutionalization of the right to leisure constitutes not only a crucial step toward building a “society of universal leisure,” but also a vital manifestation of implementing the people-centered development philosophy.

In incorporating the right to leisure into the legal framework, vigilance must be maintained against the dual risks of “nominal rights” and “rights inflation.” The legislative process should balance theoretical legitimacy, normative feasibility, and practical operability. This paper advocates prioritizing the protecting of female workers—not to exclude other groups' interests, but also adopt a phased and gradual legislative strategy based on women's particular vulnerability under pressures of reproduction and workplace demands. Future efforts should establish a multi-layered legal system, strengthen temporal security mechanisms, and enhance service support and rights remediation frameworks to form a tripartite collaborative responsibility structure engaging the state, market, and family. The ultimate objective remains achieving universal protection of all citizens' right to leisure, enabling every societal member to pursue self-realization and improved quality of life through freely disposable time.
